# Supplementary material for: Transmission of SARS‐CoV‐2 in standardised first few X cases and household transmission investigations: A systematic review and meta‐analysis
Source: Influenza Other Respir Viruses. 2022 Jun 16;16(5):803–19. doi: 10.1111/irv.13002 (PMC9343340; doi:10.1111/irv.13002)
Supplement: Supplementary file 1 — Table S1. Detailed descriptions and characteristics of investigations included in the systematic review. Table S2. Results comparing primary meta‐analysis results to those from a risk of bias subgroup meta‐analysis of household secondary infection attack rate (hSAR) after inclusion of studies at high risk of bias. I2 and τ^2 are presented for each model to indicate the percentage of variation across studies attributable to heterogeneity and the estimated between‐study variance, respectively. The p‐value from the χ2 test for heterogeneity is also presented. Table S3. Results from meta‐analyses of household secondary clinical attack rate (hSCAR). I2 and τ2 are presented for each model to indicate the percentage of variation across studies attributable to heterogeneity and the estimated between‐study variance, respectively. The p‐value from the χ2 test for heterogeneity is also presented. Figure S1: Horizontal lines showing the approximate timing of investigations reporting a household secondary infection attack rate (hSAR) by month and year. Two of the 76 investigations reporting a hSAR are not plotted as the start date was not reported. Vertical lines represent the time at which variants Alpha and Delta were designated as Variants of Concern (VoC) by the WHO (18 December 2020, and 5 May 2021, respectively). Investigations are ordered by start dates. The hSAR and 95% confidence intervals are provided on the right‐hand side. Figure S2. Results of the critical appraisal tool as applied to investigations that reported household secondary infection attack rate (hSAR). Colours for Questions 1–10 indicate whether each was addressed in the investigation (dark blue) or not (cream), or instances where there was insufficient detail available to assess (light blue). An overall rating of the risk of bias is provided in the far‐right column, with investigations rated Low (light grey), Medium (medium grey) or High (dark grey). Figure S3: Results of the critical appraisal tool as ap [file IRV-16-803-s001.docx]

Supplementary Material

**Supplementary Table 1.** Detailed descriptions and characteristics of investigations included in the systematic review.

| Author (ref) | Country | WHO Region | Income Status | HRP Status | No. Index Cases | No. Households | No. Household Contacts, Included/Total | No. Secondary Cases | Timing of Recruitment | Household Study Design | Secondary Case Ascertainment Methods(s) | Data Collection Method | Duration of Follow Up |
| --- | --- | --- | --- | --- | --- | --- | --- | --- | --- | --- | --- | --- | --- |
| Angulo-Bazan, Y. et al.^1^ | Peru | AMR | Upper-Middle | Yes | 52 | 52 | 236 | 125 [serology]; 162 [symptoms] | 23 April 2020 - 2 May 2020 | Yes | Serology [hSAR]; symptoms only [hSCAR] | Retrospective | Unclear |
| Areekal, B. et al.^2^ | India | SEAR | Lower-Middle | No | 212 | 212 | 849 | 221 [lab diagnosis]; 132 [symptoms] | June 2020 - July 2020 | No | Unclear [hSAR]; symptoms only [hSCAR] | Prospective | 14 days or less |
| Bi, Q. et al. ^3^ | China | WPR | Upper-Middle | No | 244 | Unknown | 686 | 77 | 14 January 2020 - 12 February 2020 | No | RT-PCR | Retrospective | 14 days or less |
| Boscolo-Rizzo, P. et al.^4^ | Italy | EUR | High | No | 179 | 179 | 121 [hSAR]; 296 [hSCAR] | 54 [RT-PCR]; 233 [symptoms] | March 2020 - April 2020 | Yes | RT-PCR [hSAR]; symptoms only [hSCAR] | Retrospective | Unclear |
| Buchan, S.A. et al.^5^ | Canada | AMR | High | No | 5617 | 5617 | 15597 | 3397 | 7 February 2021 - 27 February 2021 | Yes | Unclear | Prospective | 14 days or less |
| Burke, R.M. et al. (A)^6^ | United States of America | AMR | High | No | 69 | 69 | 193/201 | 18 [RT-PCR]; 62 [symptoms] | January 2020 - April 2020 | Yes | RT-PCR [hSAR]; symptoms only [hSCAR] | Prospective | 14 days or less |
| Burke, R.M. et al. (B)^7^ | United States of America | AMR | High | No | 9 | 9 | 15 | 2 [RT-PCR]; 8 [symptoms] | January 2020 | No | RT-PCR [hSAR]; symptoms only [hSCAR] | Prospective | 14 days or less |
| Carazo, S. et al.^8^ | Canada | AMR | High | No | 3823 | 3823 | 9096 | 2718 | 1 March 2020 - 14 June 2020 | No | Symptoms | Retrospective | 14 days or less |
| Cerami, C. et al.^9^ | United States of America | AMR | High | No | 100 | 100 | 176/182 | 106 [RT-PCR or serology]; 91 [symptoms] | April 2020 - October 2020 | Yes | RT-PCR or serology [hSAR]; symptoms only [hSCAR] | Prospective | Greater than 14 days |
| Chaw, L. et al.^10^ | Brunei Darussalam | WPR | High | No | 19 | 19 | 123 | 16 | March 2020 | No | RT-PCR | Prospective | 14 days or less |
| Cheng, H.Y. et al.^11^ | Taiwan, China | WPR | High | No | 100 | 100 | 151 | 10 [RT-PCR]; 7 [symptoms] | 15 January 2020 - 18 March 2020 | No | RT-PCR [hSAR]; symptoms only [hSCAR] | Prospective | 14 days or less |
| Dambadarjaa, D. et al.^12^ | Mongolia | WPR | Lower-Middle | No | 97 | 97 | 325 | 60 | November 2020 | No | RT-PCR | Prospective | 14 days or less |
| Dattner, I. et al.^13^ | Israel | EUR | High | No | 637 | 637 | 3353 | 1510 [RT-PCR]; 1243 [symptoms] | 17 March 2020 - 3 May 2020 | Yes | RT-PCR [hSAR]; symptoms only [hSCAR] | Retrospective | Unclear |
| Gomaa, M.R. et al.^14^ | Egypt | EMR | Lower-Middle | Yes | 23 | 23 | 98 | 88 [RT-PCR or serology]; 52 [symptoms] | April 2020 - October 2020 | Yes | RT-PCR or serology [hSAR]; symptoms only [hSCAR] | Prospective | 14 days or less |
| Grijalva, C.G. et al.^15^ | United States of America | AMR | High | No | 101 | 101 | 137/191 | 48 | April 2020 - September 2020 | Yes | RT-PCR | Prospective | 14 days or less |
| Heredia-Melo, D.C. et al.^16^ | Colombia | AMR | Upper-Middle | Yes | 99 | 99 | 315/334 | 140 | September 2020 - January 2021 | No | RT-PCR and symptoms | Prospective | 14 days or less |
| Hsu, C.Y. et al.^17^ | Taiwan, China | WPR | High | No | 18 | 26 | 102 | 47 | 28 January 2020 - 28 February 2021 | Yes | RT-PCR | Retrospective | 14 days or less |
| Hu, P. et al.^18^ | China | WPR | Upper-Middle | No | 82 | 82 | 267 | 46 | Before 5 March 2020 | No | RT-PCR | Retrospective | 14 days or less |
| Hu, S.X. et al.^19^ | China | WPR | Upper-Middle | No | 284 | 115 | 1021 | 99 | 23 January 2020 - 2 April 2020 | No | RT-PCR | Prospective | 14 days or less |
| Islam, F. et al.^20^ | India | SEAR | Lower-Middle | No | 99 | 99 | 254/318 | 141 | 15 December 2020 - 16 June 2021 | Yes | RT-PCR | Prospective | Greater than 14 days |
| Jashaninejad, R. et al.^21^ | Iran (Islamic Republic of) | EMR | Lower-Middle | Yes | 323 | 323 | 989 | 314 | May 2020 - July 2020 | No | RT-PCR | Prospective | 14 days or less |
| Jeewandara, C. et al.^22^ | Sri Lanka | SEAR | Lower-Middle | No | 39 | 39 | 1093 | 85 | 15 April 2020 - 19 May 2020 | No | RT-PCR | Prospective | 14 days or less |
| Jeong, E.K. et al.^23^ | Republic of Korea | WPR | High | No | 30 | 30 | 119 | 9 | 24 January 2020 - 10 March 2020 | No | Symptoms | Retrospective | 14 days or less |
| Jing, Q.L. et al.^24^ | China | WPR | Upper-Middle | No | 159 | 159 | 529 | 93 | 7 January 2020 - 18 February 2020 | Yes | RT-PCR | Retrospective | 14 days or less |
| Korean CDC^25^ | Republic of Korea | WPR | High | No | 9 | 9 | 119 | 9 | 24 January 2020 - 10 March 2020 | No | Unclear | Prospective | 14 days or less |
| Koureas, M. et al.^26^ | Greece | EUR | High | No | 30 | 30 | 164 | 62 | 8 April 2020 - 4 June 2020 | No | RT-PCR | Prospective | 14 days or less |
| Kuba, Y. et al.^27^ | Japan | WPR | High | No | 78 | 78 | 174 | 21 [RT-PCR]; 21 [symptoms] | 14 February 2020 - 31 May 2020 | Yes | RT-PCR and symptoms [hSAR]; symptoms only [hSCAR] | Prospective | 14 days or less |
| Kuwelker, K. et al.^28^ | Norway | EUR | High | No | 112 | 112 | 179/245 | 85 [RT-PCR or serology], 130 [symptoms] | 28 February 2020 - 4 April 2020 | Yes | RT-PCR or serology [hSAR]; symptoms only [hSCAR] | Prospective | Greater than 14 days |
| Laxminarayan, R. et al.^29^ | India | SEAR | Lower-Middle | No | 998 | 998 | 4065 | 380 | 5 March 2020 - 1 August 2020 | No | RT-PCR | Retrospective | 14 days or less |
| Layan, M. et al.^30^ | Israel | EUR | High | No | 215 | 210 | 687 | 269 | December 2020 - April 2021 | Yes | RT-PCR | Prospective | 14 days or less |
| Lewis, N.M. et al.^31^ | United States of America | AMR | High | No | 58 | 58 | 188/197 | 52 [RT-PCR or serology]; 136 [symptoms] | 22 March 2020 - 25 April 2020 | Yes | RT-PCR or serology [hSAR]; symptoms only [hSCAR] | Prospective | 14 days or less |
| Li, F. et al.^32^ | China | WPR | Upper-Middle | No | 24985 | 24985 | 52822 | 8447 | 2 December 2019 - 18 April 2020 | Yes | Unclear | Retrospective | 14 days or less |
| Li, W. et al.^33^ | China | WPR | Upper-Middle | No | 105 | 105 | 392 | 64 [RT-PCR]; 55 [symptoms] | 1 January 2020 - 20 February 2020 | Yes | RT-PCR [hSAR]; symptoms only [hSCAR] | Prospective | 14 days or less |
| Liu, T. et al.^34^ | China | WPR | Upper-Middle | No | 1206 | 1206 | 4707 | 410 | 10 January 2020 - 15 March 2020 | No | RT-PCR | Retrospective | 14 days or less |
| Loenenbach, A. et al.^35^ | Germany | EUR | High | No | 38 | 38 | 92 | 34 | January 2021 - February 2021 | No | RT-PCR and symptoms | Prospective | 14 days or less |
| Luo, L. et al.^36^ | China | WPR | Upper-Middle | No | 197 | 197 | 1015 | 105 | 13 January 2020 - 6 March 2020 | No | RT-PCR | Prospective | 14 days or less |
| Maltezou, H.C. et al.^37^ | Greece | EUR | High | No | 133 | 133 | 846 | 295 | 26 February 2020 - 30 June 2020 | No | RT-PCR and symptoms | Retrospective | 14 days or less |
| Marcato, A.J. et al.^38^ | Australia | WPR | High | No | 101 | 96 | 286/300 | 40 | April 2020 - October 2020 | Yes | RT-PCR | Prospective | 14 days or less |
| Marks, M. et al.^39^ | Spain | EUR | High | No | 282 | 282 | 382 | 91 | 17 March 2020 - 28 April 2020 | No | RT-PCR | Prospective | 14 days or less |
| Miller, E. et al.^40^ | United Kingdom | EUR | High | No | 117 | 117 | 248 | 75 | 30 March 2020 - 17 November 2020 | Yes | RT-PCR or serology | Prospective | 14 days or less |
| Miyahara, R. et al.^41^ | Japan | WPR | High | No | 306 | 306 | 775 | 147 | 22 February 2020 - 31 May 2020 | Yes | RT-PCR | Retrospective | Greater than 14 days |
| Mize, V. et al.^42^ | South Sudan | AFR | Low | Yes | 29 | 29 | 13089 | 1 | 8 June 2020 - 3 December 2020 | No | RT-PCR or serology | Prospective | Greater than 14 days |
| Musa, S. et al.^43^ | Bosnia and Herzegovina | EUR | Upper-Middle | No | 383 | 360 | 747 | 119 [RT-PCR and symptoms]; 103 [symptoms] | 3 August 2020 - 23 December 2020 | Yes | RT-PCR and symptoms [hSAR]; symptoms only [hSCAR] | Prospective | 14 days or less |
| Ng, O.T. et al.^44^ | Singapore | WPR | High | No | 581 | 578 | 1779/1863 | 134 [RT-PCR or serology]; 468 [symptoms] | 23 January 2020 - 3 April 2020 | Yes | RT-PCR or serology [hSAR]; symptoms only [hSCAR] | Retrospective | 14 days or less |
| Nohynek, H. et al.^45^ | Finland | EUR | High | No | 37 | 37 | 87/90 | 42 | Mar-20 | Yes | RT-PCR or serology | Prospective | Unclear |
| Park, S.Y., et al.^46^ | Republic of Korea | WPR | High | No | 97 | Unknown | 225 | 34 | 21 February 2020 - 8 March 2020 | No | RT-PCR | Prospective | 14 days or less |
| Pett, J. et al.^47^ | United Kingdom | EUR | High | No | 27 | 27 | 44 | 7 [RT-PCR]; 7 [symptoms] | 26 February 2020 - 26 April 2020 | No | RT-PCR and symptoms [hSAR]; symptoms only [hSCAR] | Prospective | 14 days or less |
| Phucharoen, C. et al.^48^ | Thailand | SEAR | Upper-Middle | No | 77 | 63 | 171 | 82 | 20 March 2020 - 2 May 2020 | No | RT-PCR | Prospective | Unclear |
| Pung, R. et al.^49^ | Singapore | WPR | High | No | 265 | 265 | 875 | 33 | Before 21 March 2020 | Yes | RT-PCR and symptoms | Prospective | 14 days or less |
| Ransom, J. et al.^50^ | South Sudan | AFR | Low | Yes | 26 | 26 | 30 [hSAR]; 42 [hSCAR] | 17 [serology]; 2 [symptoms] | Jun-20 | Yes | Serology [hSAR]; symptoms only [hSCAR] | Prospective | Unclear |
| Ratovoson, R. et al.^51^ | Madagascar | AFR | Low | No | 33 | 33 | 179/192 | 53 | 19 March 2020 - 30 July 2020 | Yes | RT-PCR or serology | Prospective | Greater than 14 days |
| Reukers, D.F.M. et al.^52^ | Netherlands | EUR | High | No | 55 | 55 | 187 | 78 [RT-PCR or serology]; 79 [symptoms] | 24 March 2020 - 6 April 2020 | Yes | RT-PCR or serology [hSAR]; symptoms only [hSCAR] | Prospective | Greater than 14 days |
| Ripabelli, G. et al.^53^ | Italy | EUR | High | No | Unknown | 34 | 59 | 16 | May-20 | No | RT-PCR | Retrospective | Greater than 14 days |
| Salihefendic, N. et al.^54^ | Bosnia and Herzegovina | EUR | Upper-Middle | No | 25 | 25 | 123 | 76 | March 2020 - December 2020 | Yes | RT-PCR or serology | Prospective | Unclear |
| Semakula, M. et al.^55^ | Rwanda | AFR | Low | Yes | 528 | 153 | 615 | 18 | 14 March 2020 - 20 July 2020 | No | RT-PCR | Retrospective | 14 days or less |
| Shah, K. et al. (A)^56^ | India | SEAR | Lower-Middle | No | 72 | 72 | 287 | 5 | March 2020 - July 2020 | Yes | Unclear | Prospective | 14 days or less |
| Shah, K. et al. (B)^57^ | India | SEAR | Lower-Middle | No | 74 | 74 | 386 | 34 | March 2020 - July 2020 | Yes | Unclear | Unclear | Greater than 14 days |
| Shambel, H. et al.^58^ | Ethiopia | AFR | Low | Yes | 100 | 100 | 221/300 | 100 | 19 May 2020 - 15 June 2020 | No | RT-PCR | Prospective | 14 days or less |
| Sharma, P. et al.^59^ | India | SEAR | Lower-Middle | No | 146 | 146 | 202/270 | 67 [RT-PCR]; 28 [symptoms] | 28 December 2020 - 28 June 2021 | Yes | RT-PCR [hSAR]; symptoms only [hSCAR] | Prospective | Greater than 14 days |
| Son, H. et al.^60^ | Republic of Korea | WPR | High | No | 108 | Unknown | 196 | 16 | 21 February 2020 - 24 March 2020 | No | RT-PCR | Prospective | 14 days or less |
| Soriano-Arandes, A. et al.^61^ | Spain | EUR | High | No | 270 | 341 | 3392 | 2091 [RT-PCR]; 1386 [symptoms] | 1 July 2020 - October 2020 | Yes | RT-PCR [hSAR]; symptoms only [hSCAR] | Prospective | Unclear |
| Sreedevi, A. et al.^62^ | India | SEAR | Lower-Middle | No | 147 | 147 | 364 | 159 [RT-PCR]; 94 [symptoms] | 21 December 2020 - 30 July 2021 | Yes | RT-PCR [hSAR]; symptoms only [hSCAR] | Prospective | Greater than 14 days |
| Sun, W.W. et al.^63^ | China | WPR | Upper-Middle | No | 149 | 149 | 697 | 240 | 8 January 2020 - 6 February 2020 | No | RT-PCR | Retrospective | 14 days or less |
| Teherani, M.F. et al.^64^ | United States of America | AMR | High | No | 32 | 32 | 144 | 67 | 16 March 2020 - 14 June 2020 | Yes | Symptoms | Prospective | 14 days or less |
| Telle, K. et al.^65^ | Norway | EUR | High | No | 7548 | 7548 | 19443 | 4613 | 1 March 2020 - 1 January 2021 | No | RT-PCR | Retrospective | 14 days or less |
| Thiel, S.L. et al.^66^ | Liechtenstein | EUR | High | No | 81 | 81 | 109/127 | 35 [serology]; 49 [symptoms] | March 2020 - April 2020 | No | Serology [hSAR]; symptoms only [hSCAR] | Prospective | 14 days or less |
| Tibebu, S. et al.^67^ | Canada | AMR | High | No | 29352 | 29352 | 84125 | 16404 | July 2020 - November 2020 | Yes | Symptoms | Prospective | Greater than 14 days |
| Tippett Barr, B. et al.^68^ | Kenya | AFR | Lower-Middle | Yes | 125 | 51 | 156 | 24 | June 2020 - October 2020 | No | RT-PCR | Prospective | 14 days or less |
| Walker, J.G. et al.^69^ | United States of America | AMR | High | No | 917 | 917 | 1766 | 655 | February 2020 - June 2020 | No | RT-PCR | Prospective | 14 days or less |
| Wang, Y. et al.^70^ | China | WPR | Upper-Middle | No | 124 | 41 | 335 | 77 | 28 February 2020 - 27 March 2020 | Yes | Unclear | Prospective | 14 days or less |
| Wang, Z. et al.^71^ | China | WPR | Upper-Middle | No | 78 | 78 | 155 | 47 [RT-PCR]; 104 [symptoms] | 13 February 2020 - 14 February 2020 | Yes | RT-PCR [hSAR]; symptoms only [hSCAR] | Retrospective | 14 days or less |
| Wei, L. et al.^72^ | China | WPR | Upper-Middle | No | 39 | 23 | 66 | 21 | January 2020 - February 2020 | Yes | Unclear | Unclear | 14 days or less |
| Wilkinson, K. et al.^73^ | Canada | AMR | High | No | 102 | 102 | 279 | 41 [RT-PCR]; 41 [symptoms] | March 2020 - 28 April 2020 | Yes | RT-PCR and symptoms [hSAR]; symptoms only [hSCAR] | Prospective | 14 days or less |
| Wu, J. et al.^74^ | China | WPR | Upper-Middle | No | 35 | 35 | 148 [hSAR]; 143 [hSCAR] | 48 [RT-PCR]; 51 [symptoms] | 17 January 2020 - 29 February 2020 | Yes | RT-PCR [hSAR]; symptoms only [hSCAR] | Prospective | Greater than 14 days |
| Wu, P. et al.^75^ | China | WPR | Upper-Middle | No | 393 | 393 | 1516 | 104 | 5 January 2020 - 7 April 2020 | No | RT-PCR | Retrospective | 14 days or less |
| Wu, Y. et al.^76^ | China | WPR | Upper-Middle | No | 144 | Unknown | 280 | 50 | 23 January 2020 - 28 February 2020 | No | Unclear | Retrospective | 14 days or less |
| Xie, W. et al.^77^ | China | WPR | Upper-Middle | No | 20 | 20 | 79 | 53 [RT-PCR]; 49 [symptoms] | 21 January 2020 - 14 February 2020 | Yes | RT-PCR [hSAR]; symptoms only [hSCAR] | Prospective | 14 days or less |
| Yadav, K. et al.^78^ | India | SEAR | Lower-Middle | No | 148 | 148 | 645 | 197 [RT-PCR or serology]; 60 [symptoms] | 16 December 2020 - 24 June 2021 | Yes | RT-PCR or serology [hSAR]; symptoms only [hSCAR] | Prospective | Greater than 14 days |
| Yi, B. et al.^79^ | China | WPR | Upper-Middle | No | 214 | 214 | 475 | 204 [RT-PCR and symptoms]; 224 [symptoms] | 22 January 2020 - 4 February 2020 | Yes | RT-PCR and symptoms [hSAR]; symptoms only [hSCAR] | Retrospective | 14 days or less |
| Yi, C. et al.^80^ | China | WPR | Upper-Middle | No | 157 | 157 | 279 | 37 [RT-PCR]; 27 [symptoms] | January 2020 - March 2020 | No | RT-PCR [hSAR]; symptoms only [hSCAR] | Prospective | 14 days or less |
| Abbreviations: ref, Reference; WHO, World Health Organization; HRP, Humanitarian Response Plan; AFR, Africa; AMR, Americas; EMR, Eastern Mediterranean; EUR, Europe; SEAR, South East Asia; WPR, Western Pacific; hSAR, household secondary infection attack rate; hSCAR, household secondary clinical attack rate; RT-PCR, reverse transcription polymerase chain reaction. | | | | | | | | | | | | | |

**
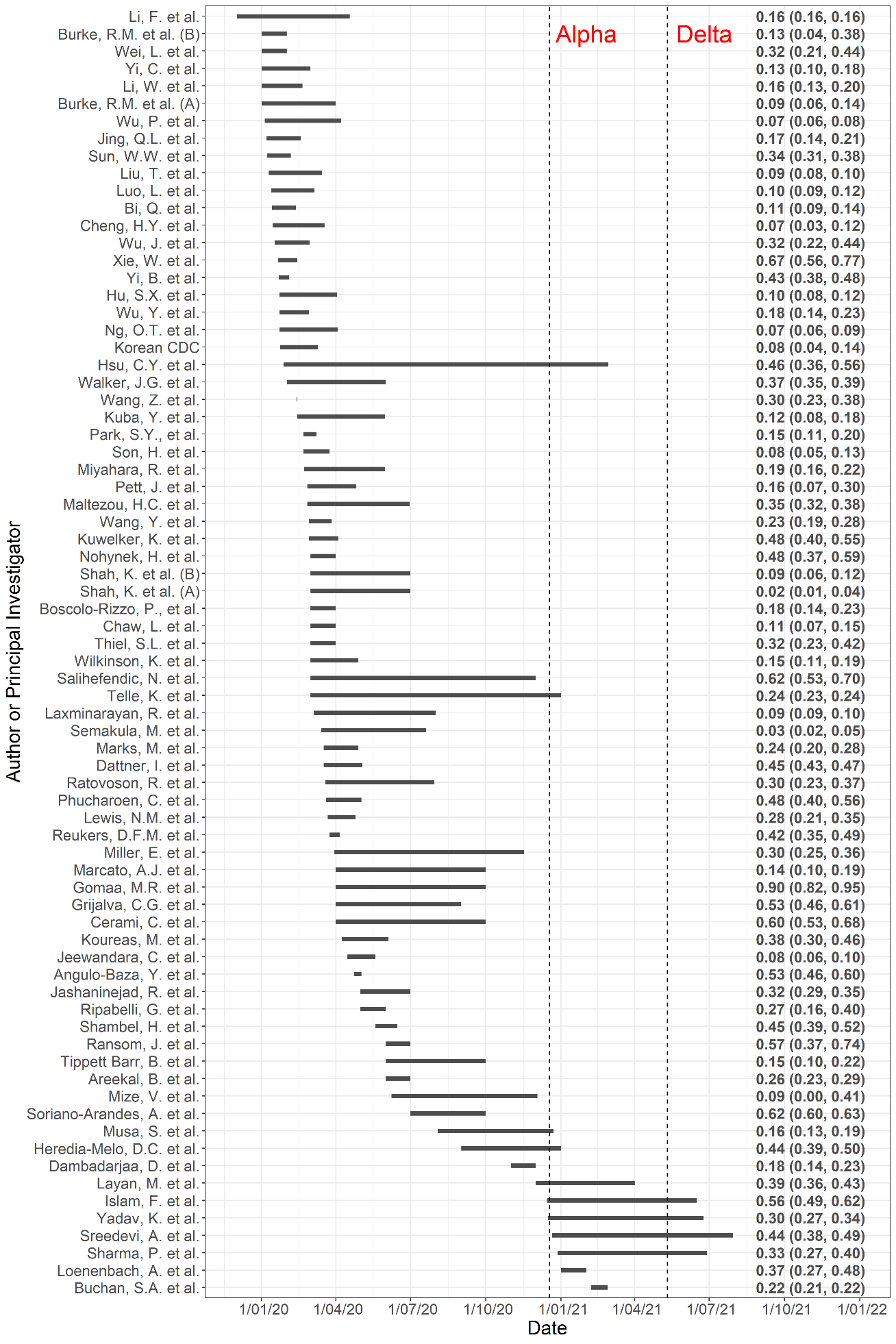
**

**Supplementary Figure 1**: Horizontal lines showing the approximate timing of investigations reporting a household secondary infection attack rate (hSAR) by month and year. Two of the 76 investigations reporting a hSAR are not plotted as the start date was not reported. Vertical lines represent the time at which variants Alpha and Delta were designated as Variants of Concern (VoC) by the WHO (18 December 2020, and 5 May 2021, respectively). Investigations are ordered by start dates. The hSAR and 95% confidence intervals are provided on the right-hand side.


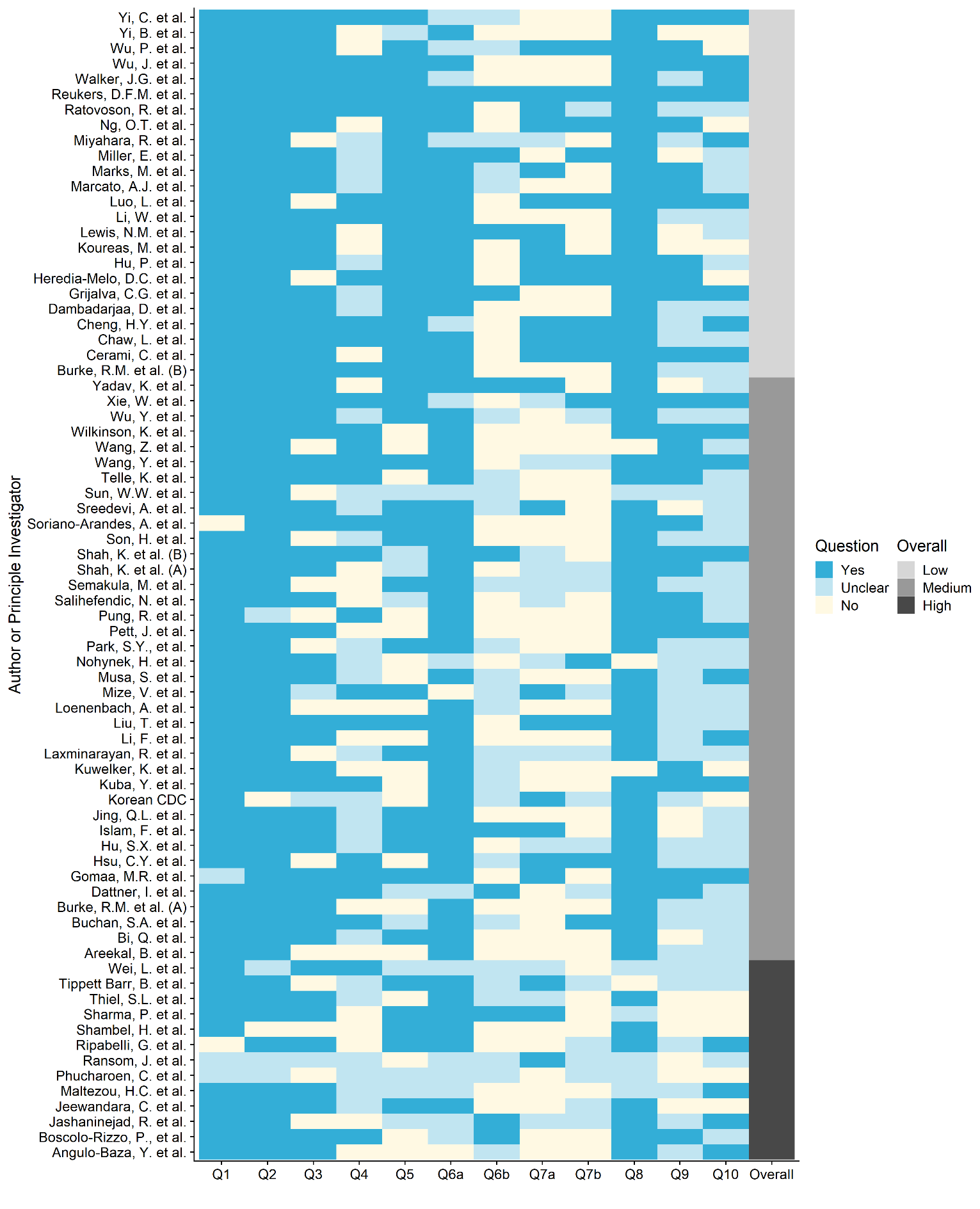


**Supplementary Figure 2.** Results of the critical appraisal tool as applied to investigations that reported household secondary infection attack rate (hSAR). Colours for Questions 1–10 indicate whether each was addressed in the investigation (dark blue) or not (cream), or instances where there was insufficient detail available to assess (light blue). An overall rating of the risk of bias is provided in the far-right column, with investigations rated Low (light grey), Medium (medium grey) or High (dark grey).


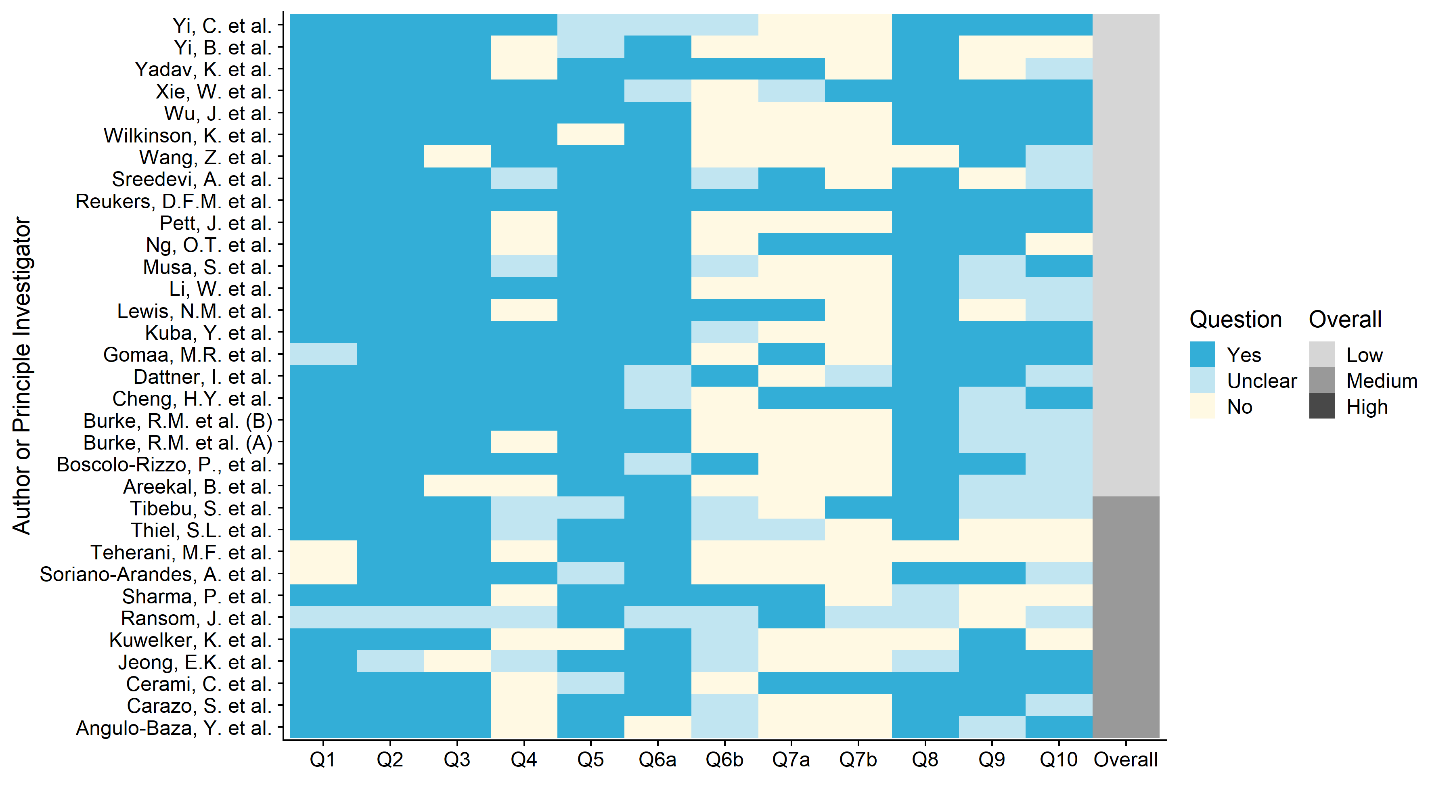


**Supplementary Figure 3:** Results of the critical appraisal tool as applied to investigations that reported household secondary clinical attack rate (hSCAR). Colours for Questions 1–10 indicate whether each was addressed in the investigation (dark blue) or not (cream), or instances where there was insufficient detail available to assess (light blue). An overall rating of the risk of bias is provided in the far-right column, with investigations rated Low (light grey), Medium (medium grey) or High (dark grey).

**
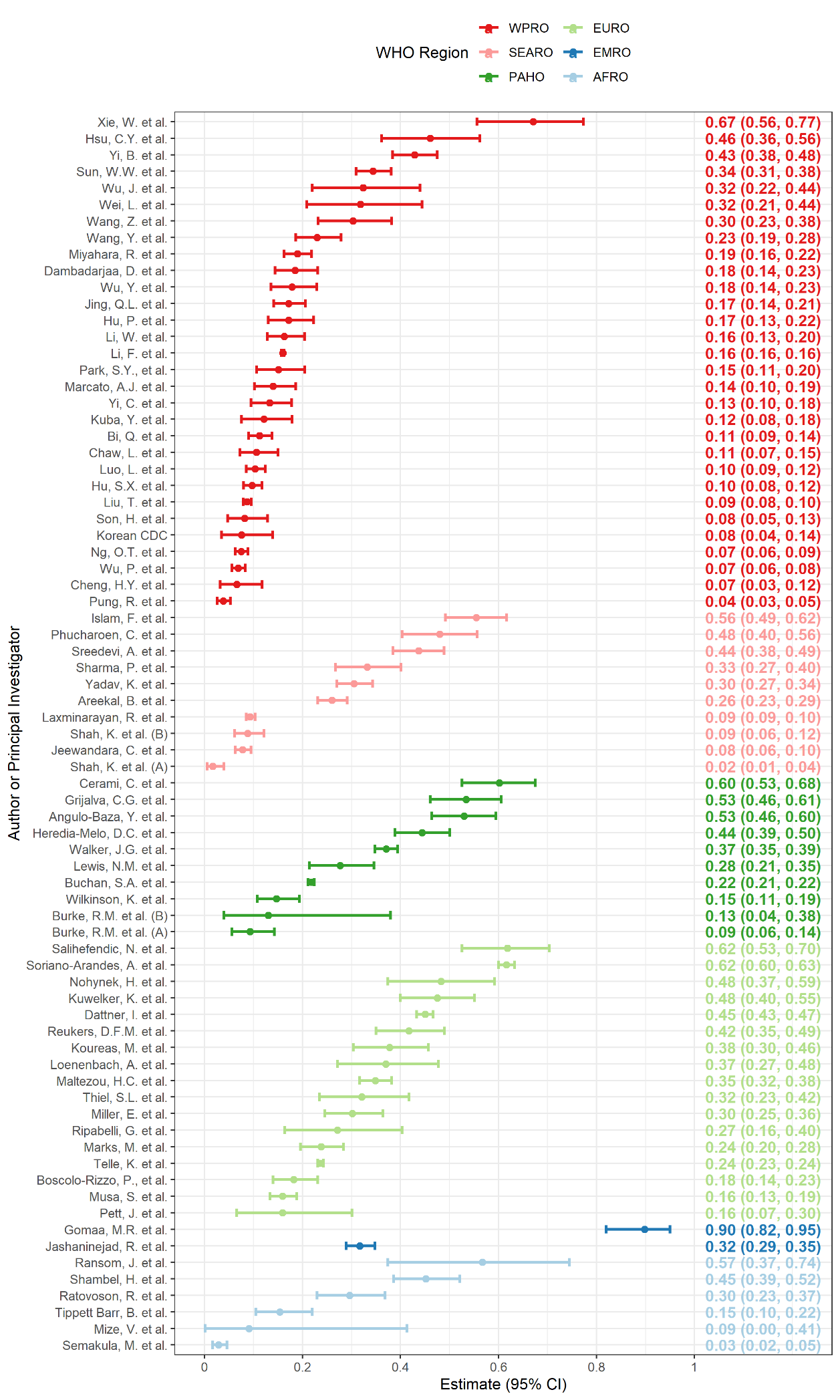
**

**Supplementary Figure 4.** Forest plot of estimated household secondary infection attack rates (hSAR) coloured by WHO Region. The estimated hSAR and 95% confidence interval are shown on the right margin.


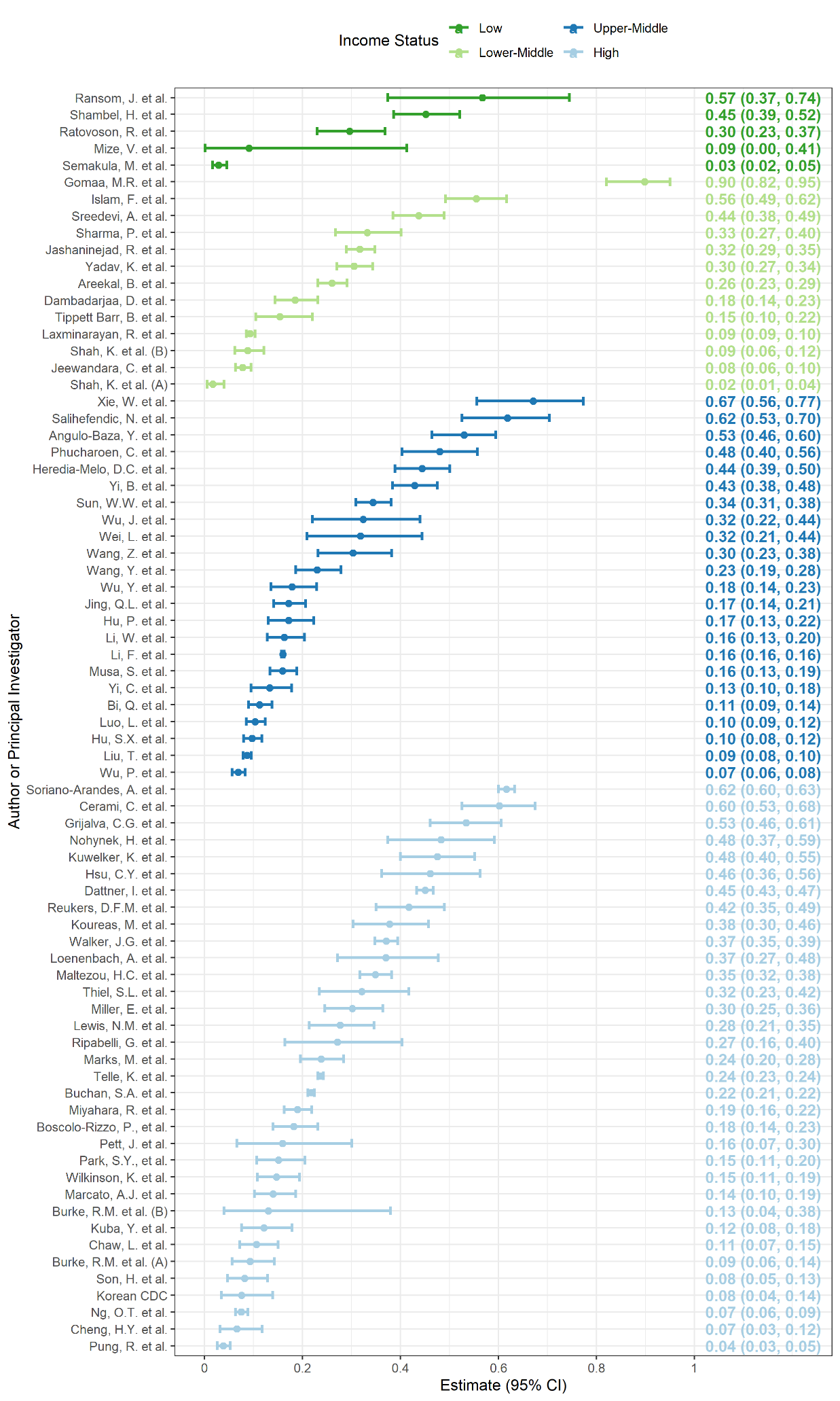


**Supplementary Figure 5.** Forest plot of estimated household secondary infection attack rates (hSAR) coloured by income status as reported by the World Bank in 2021.^81^ The estimated hSAR and 95% confidence interval are shown on the right margin.


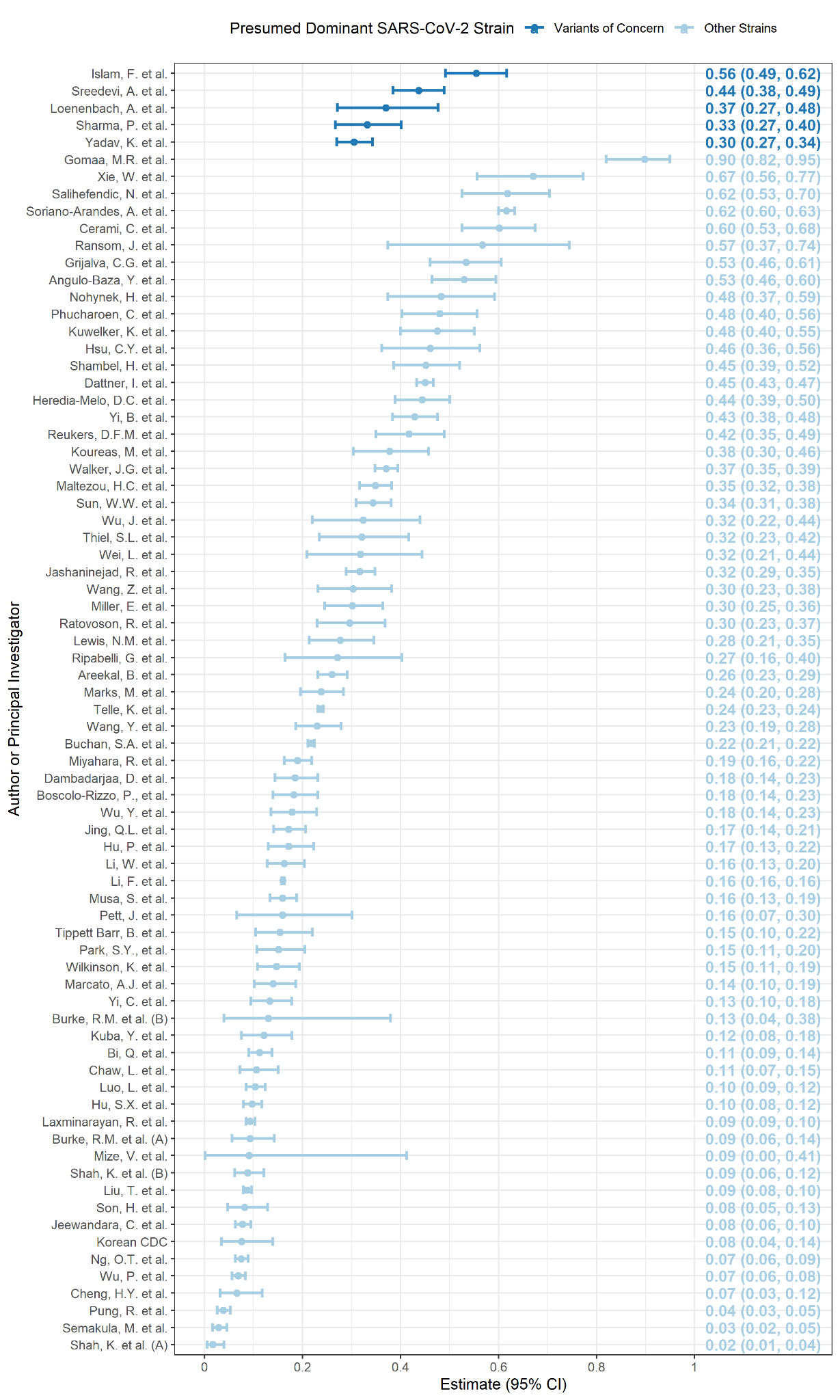


**Supplementary Figure 6.** Forest plot of estimated household secondary infection attack rates (hSAR) coloured by presumed dominant SARS-CoV-2 strain as determined by data available from CoVariants^82^ GISAID.^83^ The estimated hSAR and 95% confidence interval are shown on the right margin.

**
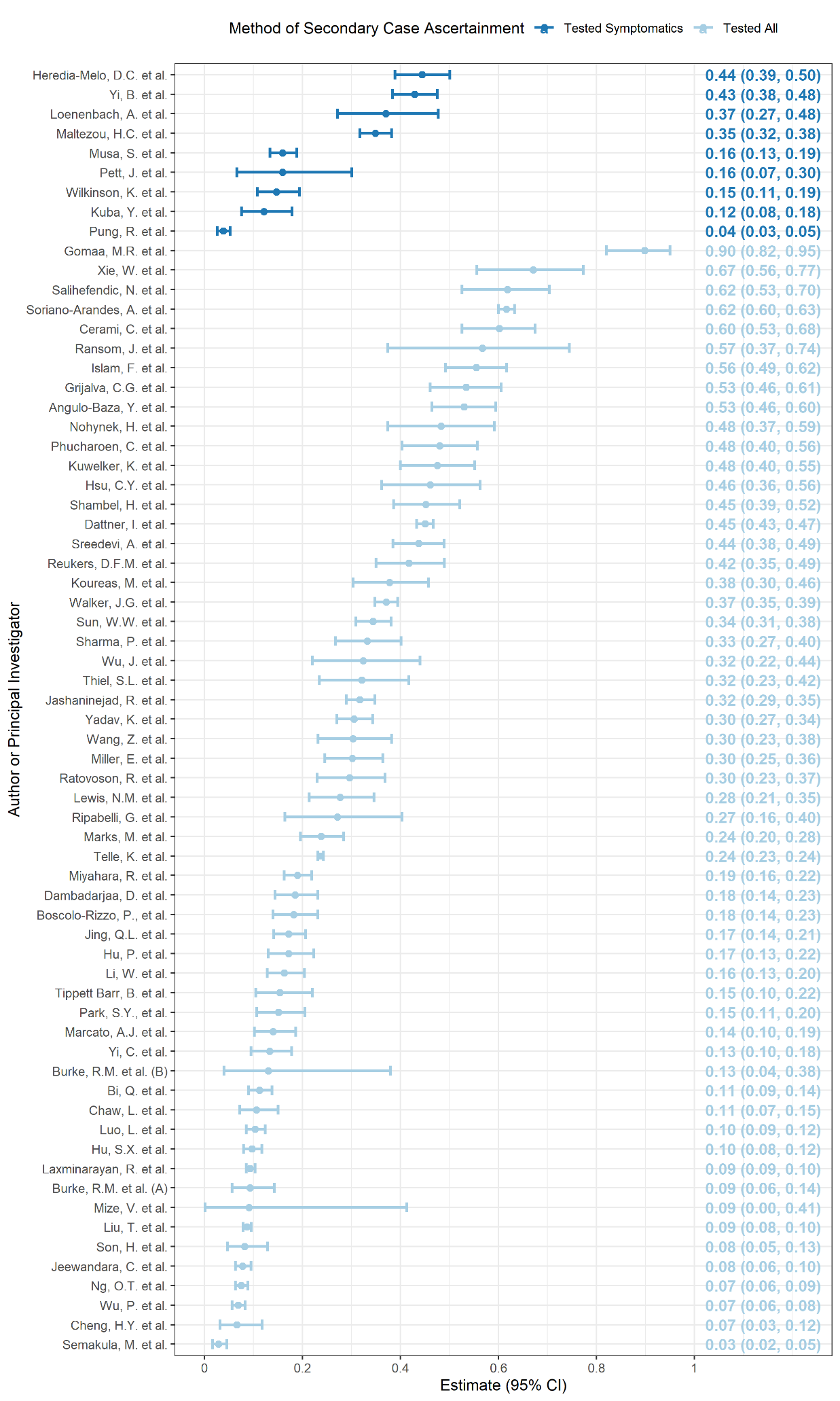
**

**Supplementary Figure 7.** Forest plot of estimated household secondary infection attack rates (hSAR) coloured by household contact testing protocol implementation. The estimated hSAR and 95% confidence interval are shown on the right margin.

**
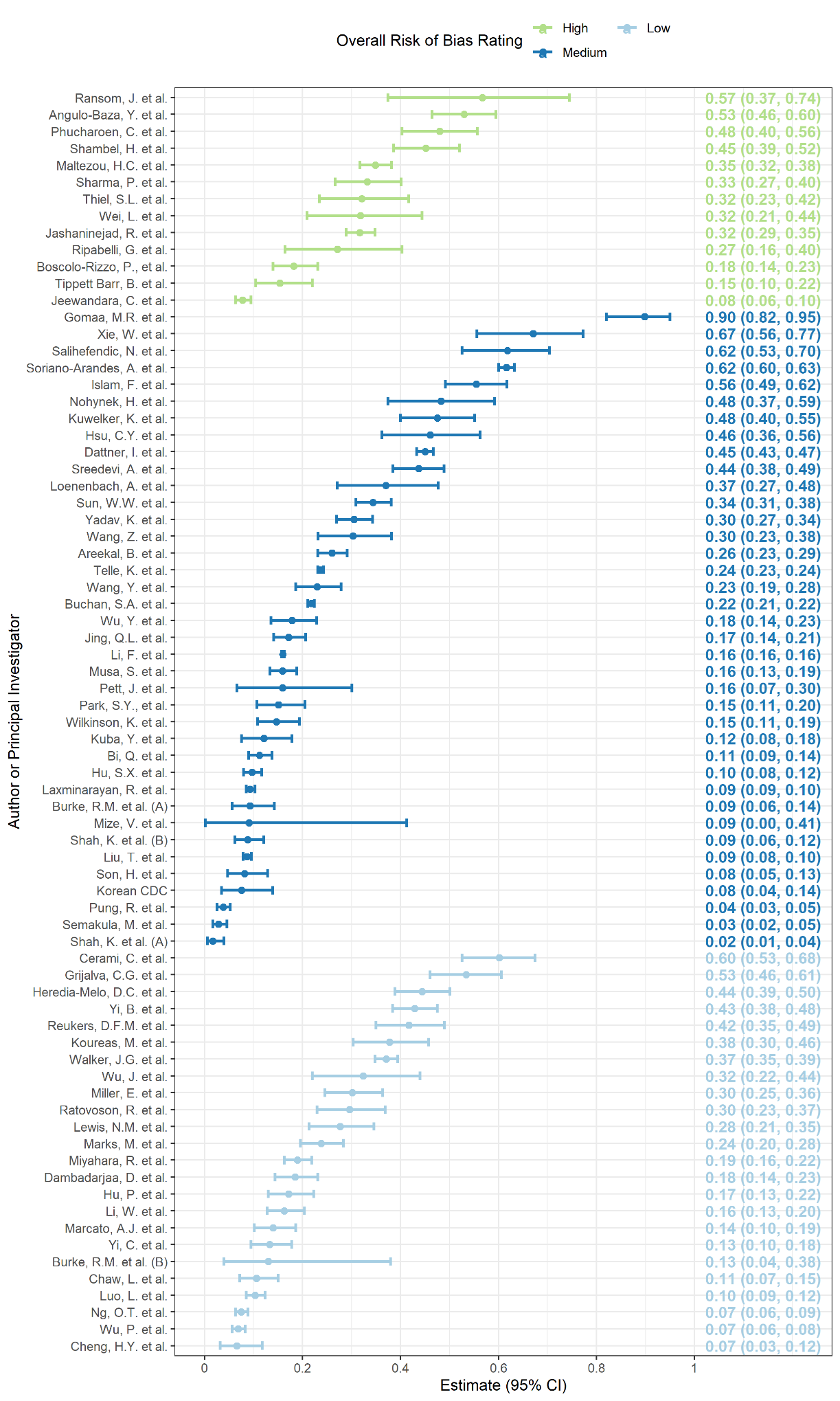
**

**Supplementary Figure 8.** Forest plot of estimated household secondary infection attack rates (hSAR) coloured by overall risk of bias assessment. The estimated hSAR and 95% confidence interval are shown on the right margin.

**
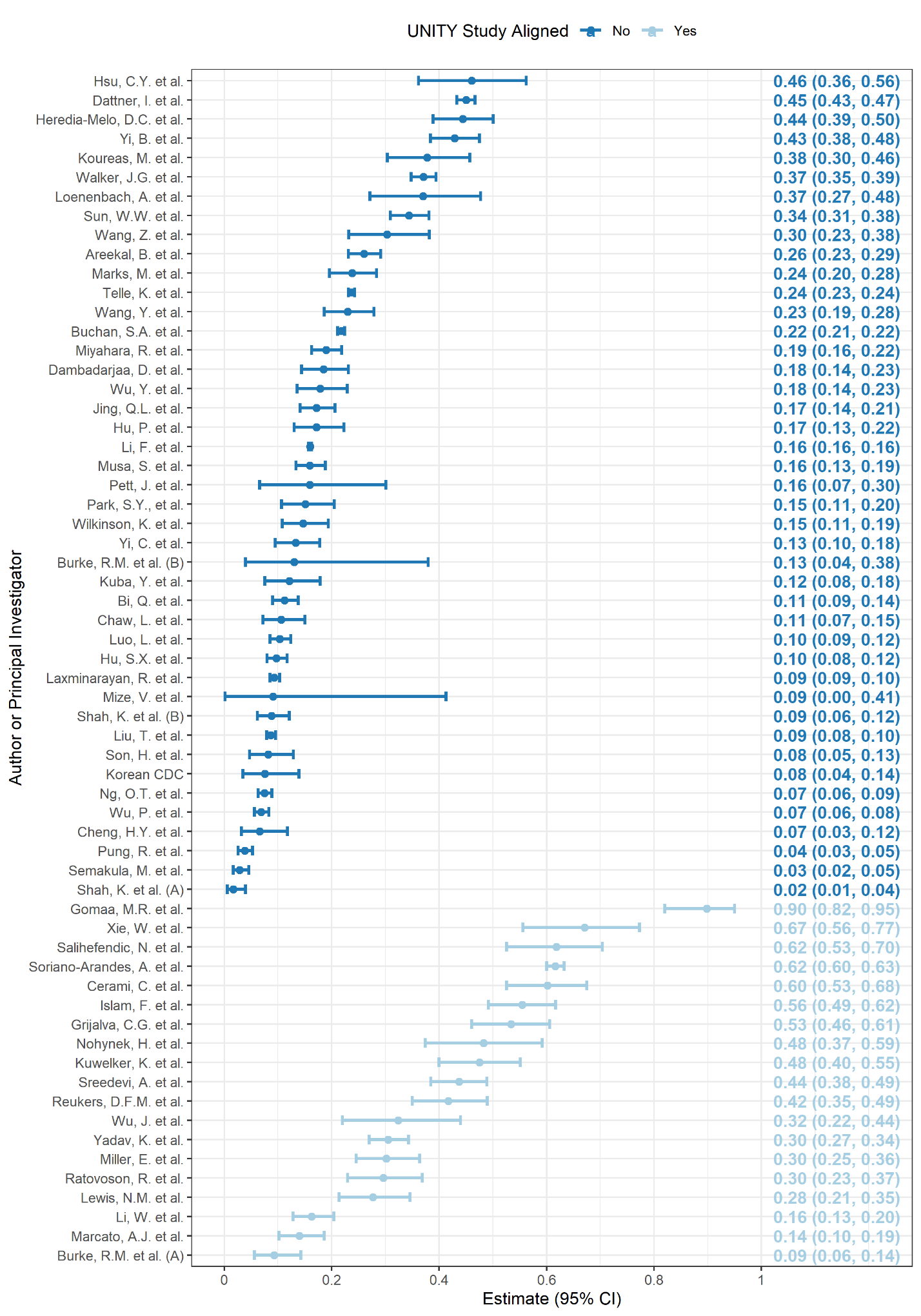
**

**Supplementary Figure 9.** Forest plot of estimated household secondary infection attack rates (hSAR) coloured by alignment to the UNITY protocol, as determined by household transmission study design, prospective data collection and routine testing of all household contacts. The estimated hSAR and 95% confidence interval are shown on the right margin.

**
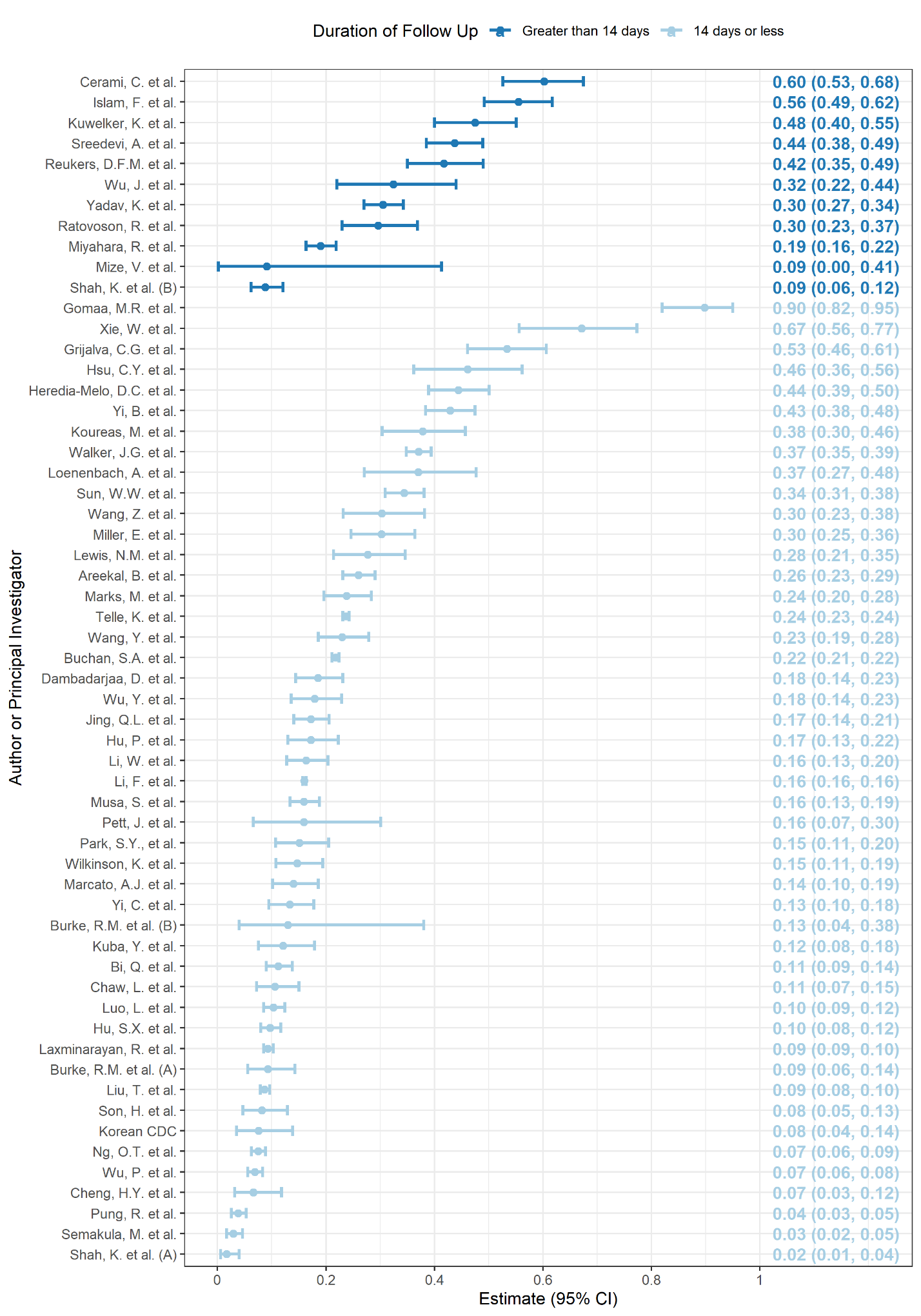
**

**Supplementary Figure 10.** Forest plot of estimated household secondary infection attack rates (hSAR) coloured by duration of follow up of household contacts. The estimated hSAR and 95% confidence interval are shown on the right margin.

**
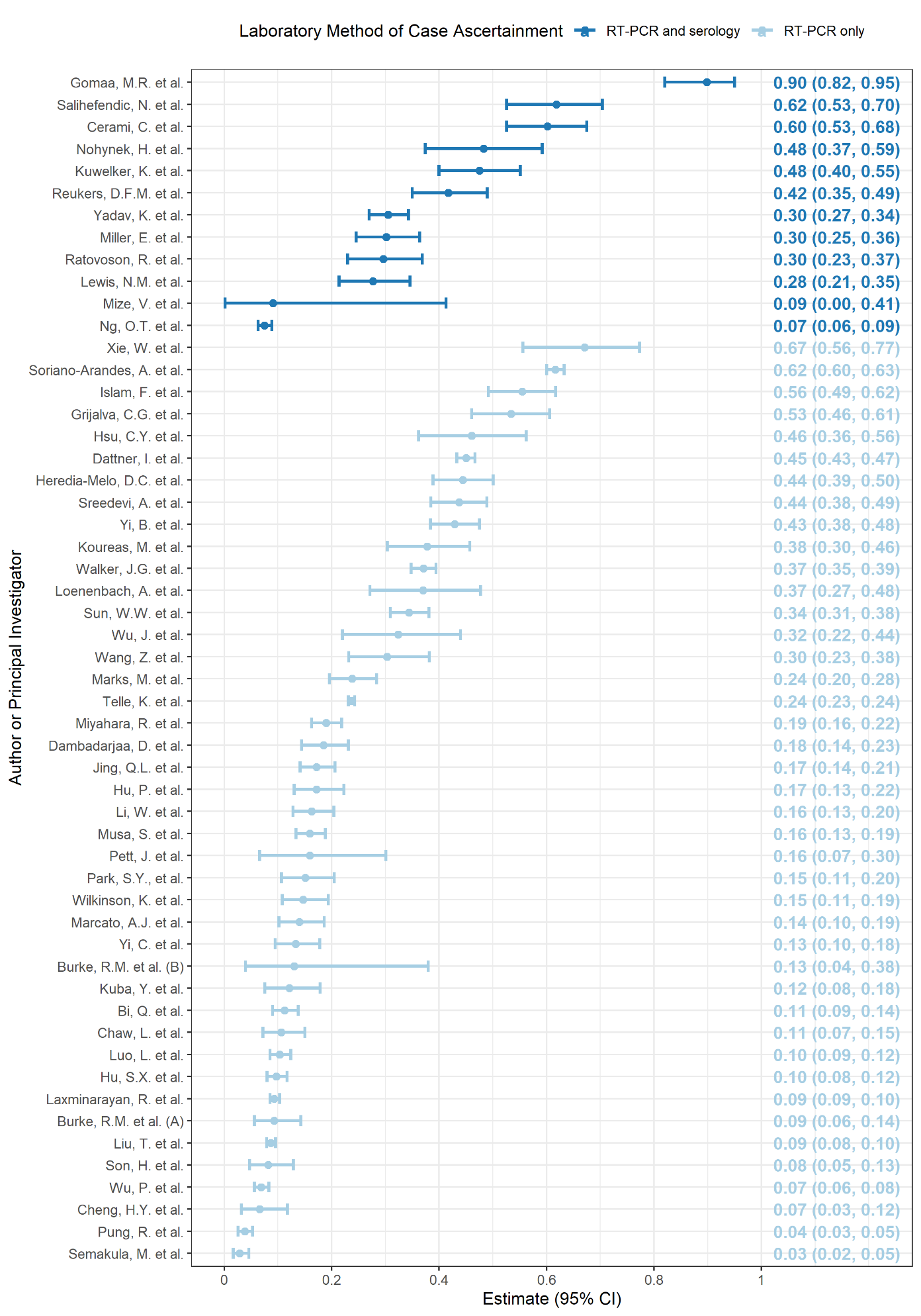
**

**Supplementary Figure 11.** Forest plot of estimated household secondary infection attack rates (hSAR) coloured by laboratory method of secondary case ascertainment. The estimated hSAR and 95% confidence interval are shown on the right margin.

**Supplementary Table 2.** Results comparing primary meta-analysis results to those from a risk of bias subgroup meta-analysis of household secondary infection attack rate (hSAR) after inclusion of studies at high risk of bias. I^2^ and τ^2^ are presented for each model to indicate the percentage of variation across studies attributable to heterogeneity and the estimated between-study variance, respectively. The p-value from the χ^2^ test for heterogeneity is also presented.

|  | **No. studies** | I^2^ | $\hat{\tau^{2}}$ | P-value |
| --- | --- | --- | --- | --- |
| Infection household secondary attack rate | 62 | 99.7 | 1.190 | <0.0001 |
| **Pre-Specified Subgroup Analyses** | | | | |
| Risk of bias assessment | 75 | 99.6 | 1.069 | <0.0001 |
| *Low or moderate risk of bias* | 62 |  | | |
| *High risk of bias* | 13 |  |  |  |

**Supplementary Table 3**. Results from meta-analyses of household secondary clinical attack rate (hSCAR). I^2^ and τ^2^ are presented for each model to indicate the percentage of variation across studies attributable to heterogeneity and the estimated between-study variance, respectively. The p-value from the χ^2^ test for heterogeneity is also presented.

|  | **No. studies** | I^2^ | $\hat{\tau^{2}}$ | P-value |
| --- | --- | --- | --- | --- |
| Clinical household secondary attack rate | 33 | 99.7 | 1.344 | <0.0001 |
| **Pre-Specified Subgroup Analyses** | | | | |
| Income Status | 33 | 99.7 | 1.324 | <0.0001 |
| *High income* | 19 |  | | |
| *Low- and middle- income* | 14 |  |  |  |
| Predominant circulating strain | 33 | 99.7 | 1.249 | <0.0001 |
| *Other strain* | 30 |  | | |
| *Variant of concern* | 3 |  |  |  |

**
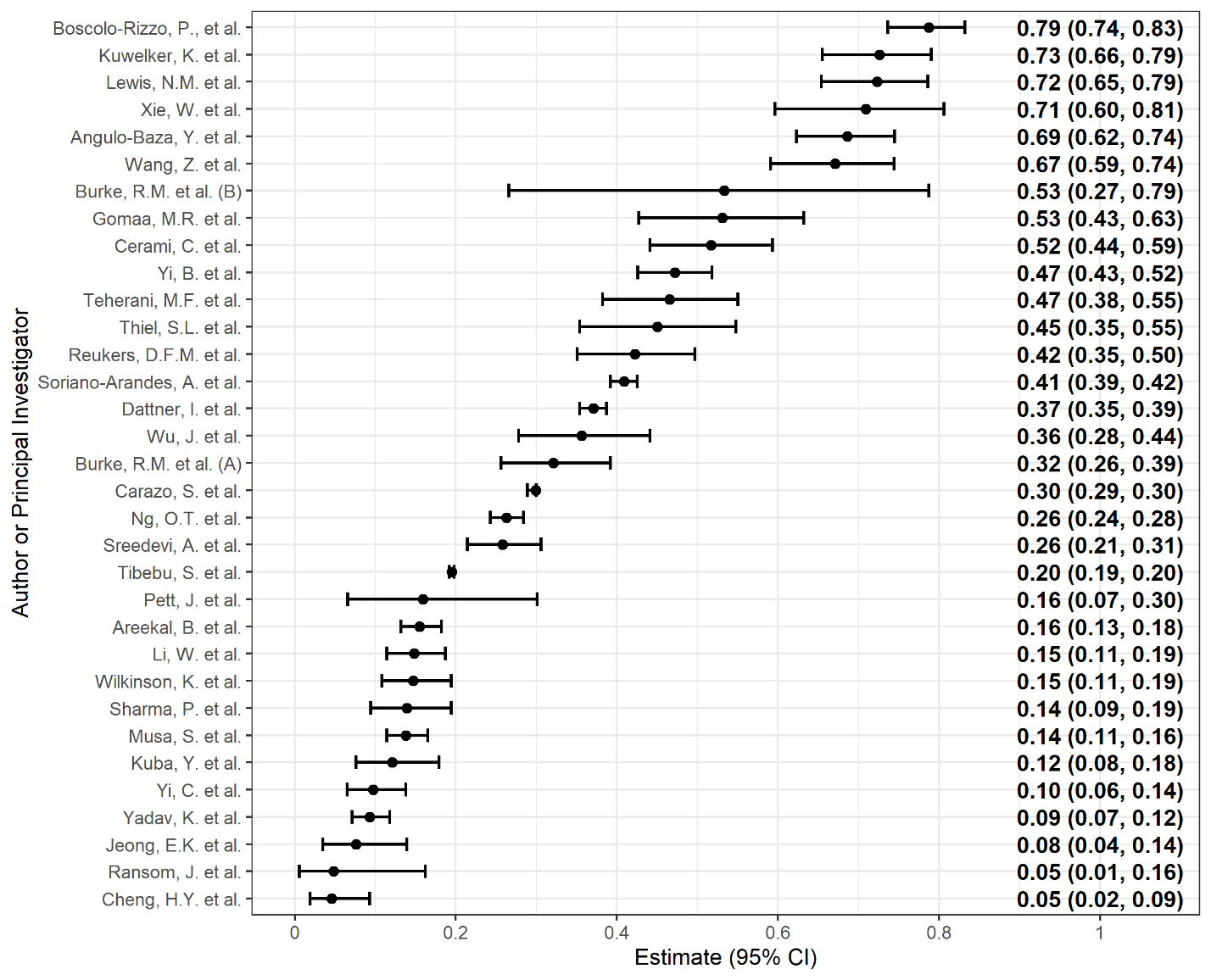
**

**Supplementary Figure 12**. Forest plot of the household secondary clinical attack rates (hSCAR) in included articles (n = 33), ordered from highest estimated hSCAR (top) to lowest estimated hSCAR (bottom). The hSAR and 95% confidence intervals (CI) are shown on the right margin.


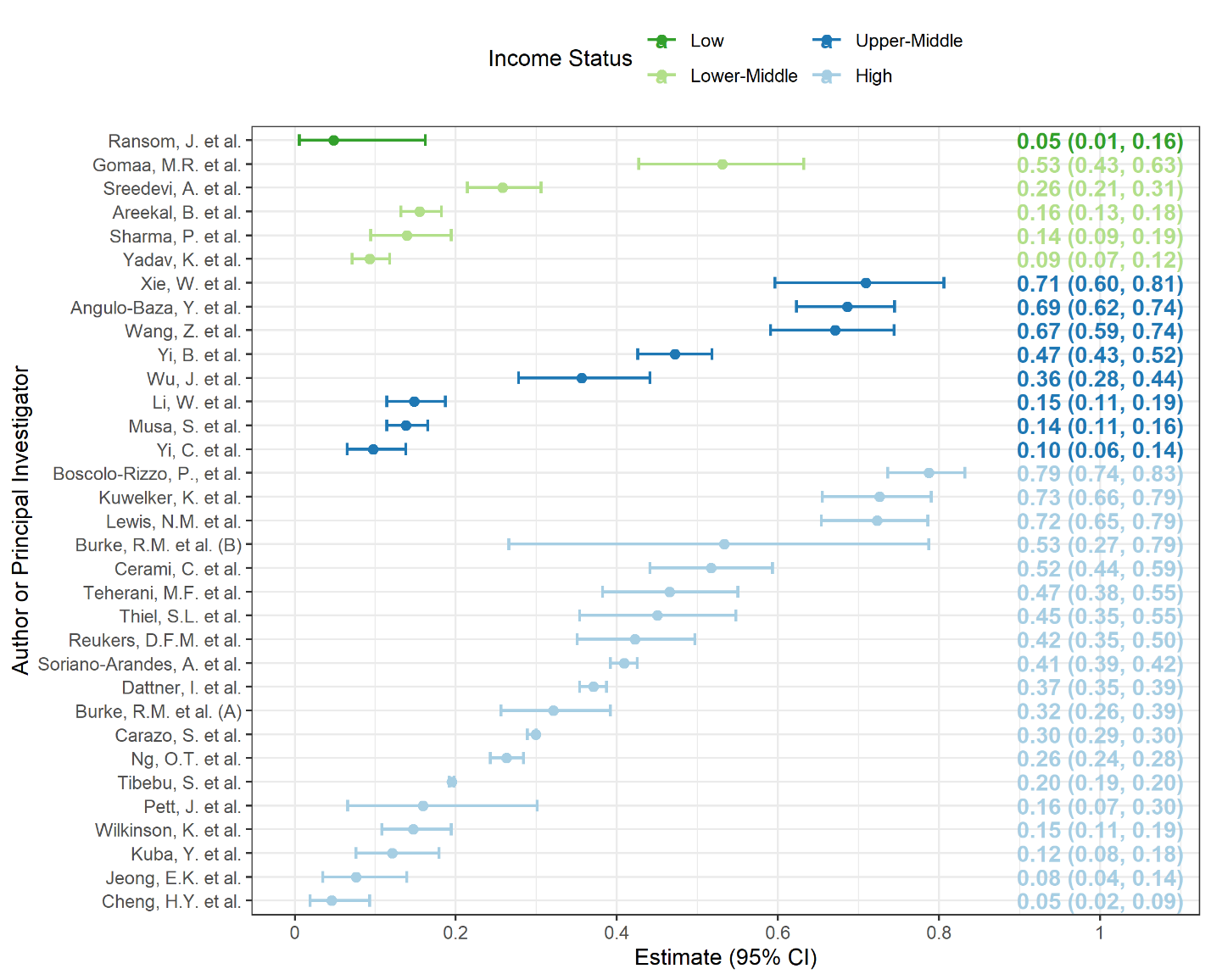


**Supplementary Figure 13.** Forest plot of estimated household secondary clinical attack rates (hSCAR) coloured by income status as reported by the World Bank in 2021.^81^ The estimated hSCAR and 95% confidence interval are shown on the right margin.


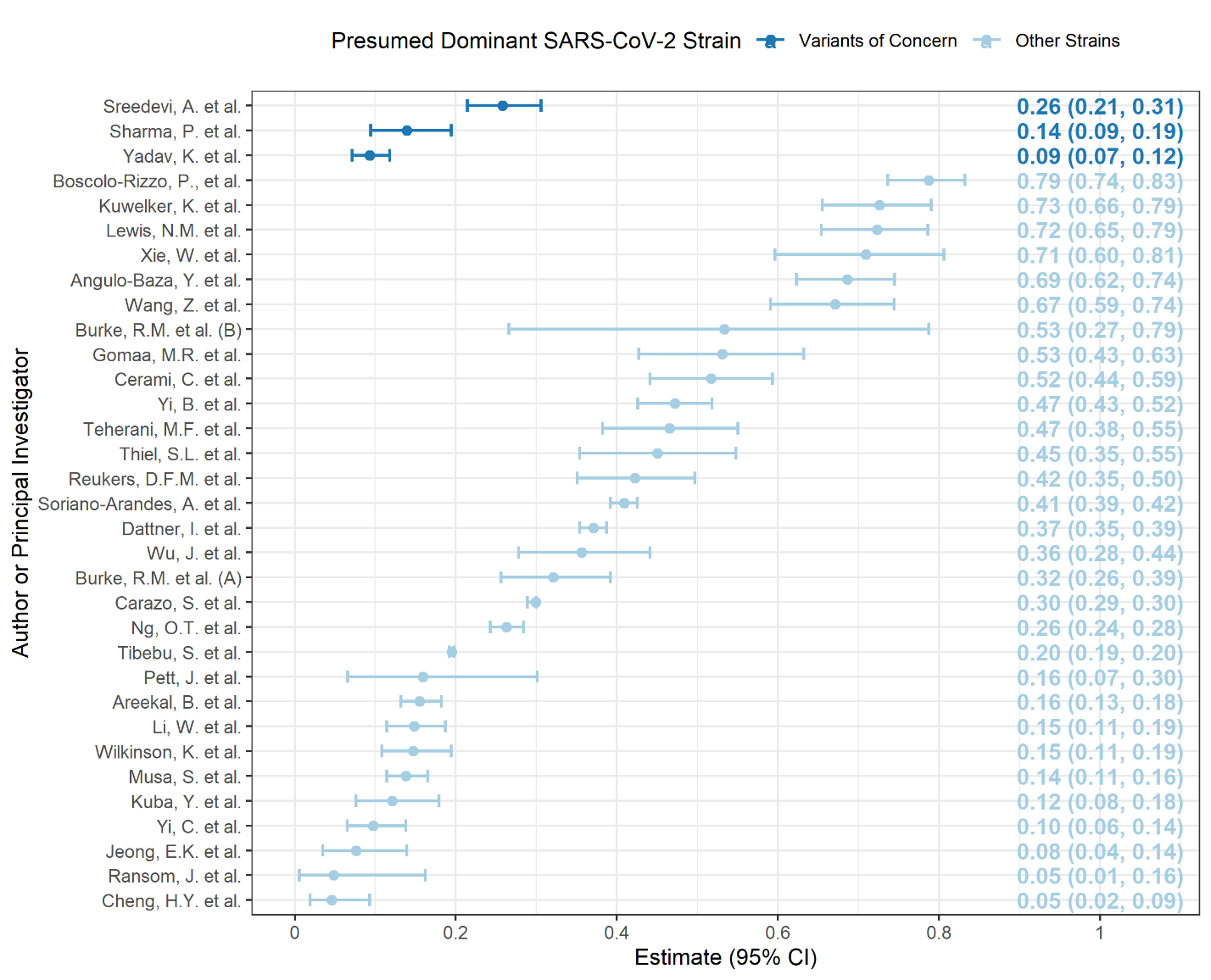


**Supplementary Figure 14.** Forest plot of estimated household secondary clinical attack rates (hSCAR) coloured by presumed dominant SARS-CoV-2 strain as determined by data available from CoVariants^82^ and GISAID^83^. The estimated hSCAR and 95% confidence interval are shown on the right margin.

References

1. Angulo-Bazan Y, Solis-Sanchez G, Cardenas F, Jorge A, Acosta J, Cabezas C. Household transmission of SARS-CoV-2 (COVID-19) in Lima, Peru. *Cadernos de saude publica*. 2021;37(3):e00238720. doi:<https://dx.doi.org/10.1590/0102-311X00238720>

2. Areekal B, Vijayan SM, Suseela MS, et al. Risk factors, epidemiological and clinical outcome of close contacts of covid-19 cases in a tertiary hospital in southern india. *Journal of Clinical and Diagnostic Research*. 2021;15(3):LC34-LC37. doi:<http://dx.doi.org/10.7860/JCDR/2021/48059.14664>

3. Bi Q, Wu Y, Mei S, et al. Epidemiology and transmission of COVID-19 in 391 cases and 1286 of their close contacts in Shenzhen, China: a retrospective cohort study. *The Lancet Infectious diseases*. 2020;20(8):911-919. doi:<https://dx.doi.org/10.1016/S1473-3099(20)30287-5>

4. Boscolo-Rizzo P, Borsetto D, Spinato G, et al. New onset of loss of smell or taste in household contacts of home-isolated SARS-CoV-2-positive subjects. *European Archives of Oto-Rhino-Laryngology*. 2020;277(9):2637-2640. doi:10.1007/s00405-020-06066-9

5. Buchan SA, Tibebu S, Daneman N, et al. Increased household secondary attacks rates with Variant of Concern SARS-CoV-2 index cases. *Clinical infectious diseases : an official publication of the Infectious Diseases Society of America*. 2021;(a4j, 9203213)doi:<https://dx.doi.org/10.1093/cid/ciab496>

6. Burke RM, Calderwood L, Killerby ME, et al. Patterns of Virus Exposure and Presumed Household Transmission among Persons with Coronavirus Disease, United States, January-April 2020. *Emerging infectious diseases*. 2021;27(9)doi:<https://dx.doi.org/10.3201/eid2709.204577>

7. Burke RM, Balter S, Barnes E, et al. Enhanced contact investigations for nine early travel-related cases of SARS-CoV-2 in the United States. *PloS one*. 2020;15(9):e0238342. doi:<https://dx.doi.org/10.1371/journal.pone.0238342>

8. Carazo S, Dionne M, Laliberte D, et al. Characterization and evolution of infection control practices among SARS-CoV-2 infected healthcare workers of acute care hospitals and long-Term care facilities in Quebec, Canada, Spring 2020. *Infection Control and Hospital Epidemiology*. 2021;((Carazo, Dionne, De Serres) CHU de Quebec-Universite Research Center, Quebec Laval, QC, Canada(Laliberte, De Serres) Department of Social and Preventive Medicine, Laval University, Quebec, Canada(Laliberte) CIUSSS de la Capitale-Nationale, Quebec, Canada()doi:<http://dx.doi.org/10.1017/ice.2021.160>

9. Cerami C, Rapp T, Lin F-C, et al. High household transmission of SARS-CoV-2 in the United States: living density, viral load, and disproportionate impact on communities of color. *medRxiv : the preprint server for health sciences*. 2021;(101767986)doi:<https://dx.doi.org/10.1101/2021.03.10.21253173>

10. Chaw L, Koh WC, Jamaludin SA, Naing L, Alikhan MF, Wong J. Analysis of SARS-CoV-2 Transmission in Different Settings, Brunei. *EMERGING INFECTIOUS DISEASES*. 2020;26(11):2598-2606. doi:10.3201/eid2611.202263

11. Cheng H-Y, Jian S-W, Liu D-P, et al. Contact Tracing Assessment of COVID-19 Transmission Dynamics in Taiwan and Risk at Different Exposure Periods Before and After Symptom Onset. *JAMA internal medicine*. 2020;180(9):1156-1163. doi:<https://dx.doi.org/10.1001/jamainternmed.2020.2020>

12. Dambadarjaa D, Mend T, Mandakh U. Mongolia Unity FFX study for COVID-19. Zenodo; 2022.

13. Dattner I, Goldberg Y, Katriel G, et al. The role of children in the spread of COVID-19: Using household data from Bnei Brak, Israel, to estimate the relative susceptibility and infectivity of children. *PLOS COMPUTATIONAL BIOLOGY*. 2021;17(2)doi:10.1371/journal.pcbi.1008559

14. Gomaa MR, El Rifay AS, Shehata M, et al. Incidence, household transmission, and neutralizing antibody seroprevalence of Coronavirus Disease 2019 in Egypt: Results of a community-based cohort. *PLoS pathogens*. 2021;17(3):e1009413. doi:<https://dx.doi.org/10.1371/journal.ppat.1009413>

15. Grijalva CG, Rolfes MA, Zhu Y, et al. Transmission of SARS-COV-2 Infections in Households - Tennessee and Wisconsin, April-September 2020. *MMWR Morbidity and mortality weekly report*. 2020;69(44):1631-1634. doi:<https://dx.doi.org/10.15585/mmwr>. mm6944e1

16. Heredia-Melo DC, Osorio-Merchán MB, González-Duarte MA. Vigilancia Intensificada de 100 casos y contactos de Influenza y SARS CoV-2, en Meta, Tolima y Cundinamarca, Colombia, 2020. (Intensified public health surveillance for first 100 cases in Meta, Tolima, and Cundinamarca, Colombia, 2020). Zenodo; 2022.

17. Hsu C-Y, Wang J-T, Huang K-C, Fan AC-H, Yeh Y-P, Chen SL-S. Household transmission but without the community-acquired outbreak of COVID-19 in Taiwan. *Journal of the Formosan Medical Association = Taiwan yi zhi*. 2021;120 Suppl 1(9214933, blq):S38-S45. doi:<https://dx.doi.org/10.1016/j.jfma.2021.04.021>

18. Hu P, Ma M, Jing Q, et al. Retrospective study identifies infection related risk factors in close contacts during COVID-19 epidemic. *International journal of infectious diseases : IJID : official publication of the International Society for Infectious Diseases*. 2021;103(c3r, 9610933):395-401. doi:<https://dx.doi.org/10.1016/j.ijid.2020.12.011>

19. Hu SX, Wang W, Wang Y, et al. Infectivity, susceptibility, and risk factors associated with SARS-CoV-2 transmission under intensive contact tracing in Hunan, China. *NATURE COMMUNICATIONS*. 2021;12(1)doi:10.1038/s41467-021-21710-6

20. Islam F, Alvi Y, Ahmad M, et al. An epidemiological Study to Assess Household Transmission & Associated Risk Factors for COVID-19 Disease amongst Residents of Delhi, India. Zenodo; 2022.

21. Jashaninejad R, Doosti-Irani A, Karami M, Keramat F, Mirzaei M. Transmission of COVID-19 and its Determinants among Close Contacts of COVID-19 Patients. *JOURNAL OF RESEARCH IN HEALTH SCIENCES*. 2021;21(2)doi:10.34172/jrhs.2021.48

22. Jeewandara C, Guruge D, Jayathilaka D, et al. Transmission dynamics, clinical characteristics and sero-surveillance in the COVID-19 outbreak in a population dense area of Colombo, Sri Lanka April- May 2020. *PLOS ONE*. 2021;16(11):e0257548. doi:10.1371/journal.pone.0257548

23. Jeong EK, Park O, Park YJ, et al. Coronavirus disease-19: Summary of 2,370 contact investigations of the first 30 cases in the Republic of Korea. *Osong Public Health and Research Perspectives*. 2020;11(2):81-84. doi:<http://dx.doi.org/10.24171/j.phrp.2020.11.2.04>

24. Jing Q-L, Liu M-J, Zhang Z-B, et al. Household secondary attack rate of COVID-19 and associated determinants in Guangzhou, China: a retrospective cohort study. *The Lancet Infectious diseases*. 2020;20(10):1141-1150. doi:<https://dx.doi.org/10.1016/S1473-3099(20)30471-0>

25. Choe YJ. Coronavirus disease-19: Summary of 2,370 Contact Investigations of the First 30 Cases in the Republic of Korea. 2020;

26. Koureas M, Speletas M, Bogogiannidou Z, et al. Transmission Dynamics of SARS-CoV-2 during an Outbreak in a Roma Community in Thessaly, Greece-Control Measures and Lessons Learned. *International journal of environmental research and public health*. 2021;18(6)doi:<https://dx.doi.org/10.3390/ijerph18062878>

27. Kuba Y, Shingaki A, Nidaira M, et al. The characteristics of household transmission during COVID-19 outbreak in Okinawa, Japan from February to May 2020. *Japanese journal of infectious diseases*. 2021;(dii, 100893704)doi:<https://dx.doi.org/10.7883/yoken.JJID.2020.943>

28. Kuwelker K, Zhou F, Blomberg B, et al. Attack rates amongst household members of outpatients with confirmed COVID-19 in Bergen, Norway: A case-ascertained study. *The Lancet regional health Europe*. 2021;3(101777707):100014. doi:<https://dx.doi.org/10.1016/j.lanepe.2020.100014>

29. Laxminarayan R, Wahl B, Dudala SR, et al. Epidemiology and transmission dynamics of COVID-19 in two Indian states. *SCIENCE*. 2020;370(6517):691-+. doi:10.1126/science.abd7672

30. Layan M, Gilboa M, Gonen T, et al. Impact of BNT162b2 vaccination and isolation on SARS-CoV-2 transmission in Israeli households: an observational study. *medRxiv*. 2021:2021.07.12.21260377. doi:10.1101/2021.07.12.21260377

31. Lewis NM, Chu VT, Ye D, et al. Household Transmission of SARS-CoV-2 in the United States. *Clinical infectious diseases : an official publication of the Infectious Diseases Society of America*. 2020;(a4j, 9203213)doi:<https://dx.doi.org/10.1093/cid/ciaa1166>

32. Li F, Li Y-Y, Liu M-J, et al. Household transmission of SARS-CoV-2 and risk factors for susceptibility and infectivity in Wuhan: a retrospective observational study. *The Lancet Infectious diseases*. 2021;21(5):617-628. doi:<https://dx.doi.org/10.1016/S1473-3099(20)30981-6>

33. Li W, Zhang B, Lu J, et al. Characteristics of Household Transmission of COVID-19. *Clinical infectious diseases : an official publication of the Infectious Diseases Society of America*. 2020;71(8):1943-1946. doi:<https://dx.doi.org/10.1093/cid/ciaa450>

34. Liu T, Liang WJ, Zhong HJ, et al. Risk factors associated with COVID-19 infection: a retrospective cohort study based on contacts tracing. *EMERGING MICROBES & INFECTIONS*. 2020;9(1):1546-1553. doi:10.1080/22221751.2020.1787799

35. Loenenbach A, Markus I, Lehfeld A-S, et al. SARS-CoV-2 variant B.1.1.7 susceptibility and infectiousness of children and adults deduced from investigations of childcare centre outbreaks, Germany, 2021. *Euro surveillance : bulletin Europeen sur les maladies transmissibles = European communicable disease bulletin*. 2021;26(21)doi:<https://dx.doi.org/10.2807/1560-7917.ES.2021.26.21.2100433>

36. Luo L, Liu D, Liao X, et al. Contact Settings and Risk for Transmission in 3410 Close Contacts of Patients With COVID-19 in Guangzhou, China : A Prospective Cohort Study. *Annals of internal medicine*. 2020;173(11):879-887. doi:<https://dx.doi.org/10.7326/M20-2671>

37. Maltezou HC, Magaziotou I, Dedoukou X, et al. Children and Adolescents With SARS-CoV-2 Infection: Epidemiology, Clinical Course and Viral Loads. *The Pediatric infectious disease journal*. 2020;39(12):e388-e392. doi:<https://dx.doi.org/10.1097/INF.0000000000002899>

38. Marcato AJ, Black AJ, Walker J, et al. Learnings from the Australian First Few X Household Transmission Project for COVID-19. *medRxiv*. 2022:2022.01.23.22269031. doi:10.1101/2022.01.23.22269031

39. Marks M, Millat-Martinez P, Ouchi D, et al. Transmission of COVID-19 in 282 clusters in Catalonia, Spain: a cohort study. *The Lancet Infectious diseases*. 2021;21(5):629-636. doi:<https://dx.doi.org/10.1016/S1473-3099(20)30985-3>

40. Miller E, Waight PA, Andrews NJ, et al. Transmission of SARS-CoV-2 in the household setting: A prospective cohort study in children and adults in England. *J Infect*. Oct 2021;83(4):483-489. doi:10.1016/j.jinf.2021.07.037

41. Miyahara R, Tsuchiya N, Yasuda I, et al. Familial Clusters of Coronavirus Disease in 10 Prefectures, Japan, February-May 2020. *Emerging infectious diseases*. 2021;27(3):915-918. doi:<https://dx.doi.org/10.3201/eid2703.203882>

42. Mize V, Meagher N, Wamala J. South Sudan FFX Unity study for COVID-19. Zenodo; 2022.

43. Musa S, Kissling E, Valenciano M, et al. Household transmission of SARS-CoV-2: a prospective observational study in Bosnia and Herzegovina, August–December 2020. *International Journal of Infectious Diseases*. 2021/11/01/ 2021;112:352-361. doi:<https://doi.org/10.1016/j.ijid.2021.09.063>

44. Ng OT, Marimuthu K, Koh V, et al. SARS-CoV-2 seroprevalence and transmission risk factors among high-risk close contacts: a retrospective cohort study. *The Lancet Infectious diseases*. 2021;21(3):333-343. doi:<https://dx.doi.org/10.1016/S1473-3099(20)30833-1>

45. Nohynek H, Melin M, Solastie A, Dub T. Finland Household Unity study for COVID-19. Zenodo; 2022.

46. Park SY, Kim Y-M, Yi S, et al. Coronavirus Disease Outbreak in Call Center, South Korea. *Emerging infectious diseases*. 2020;26(8):1666-1670. doi:<https://dx.doi.org/10.3201/eid2608.201274>

47. Pett J, McAleavey P, McGurnaghan P, et al. Epidemiology of COVID-19 in Northern Ireland, 26 February 2020-26 April 2020. *Epidemiology and infection*. 2021;149(epi, 8703737):e36. doi:<https://dx.doi.org/10.1017/S0950268821000224>

48. Phucharoen C, Sangkaew N, Stosic K. The characteristics of COVID-19 transmission from case to high-risk contact, a statistical analysis from contact tracing data. *ECLINICALMEDICINE*. 2020;27doi:10.1016/j.eclinm.2020.100543

49. Pung R, Park M, Cook AR, Lee VJ. Age-related risk of household transmission of COVID-19 in Singapore. *Influenza and other respiratory viruses*. 2021;15(2):206-208. doi:<https://dx.doi.org/10.1111/irv.12809>

50. Ransom J, Lako R, Dulacha D, Joseph W, Olu OO. South Sudan Household Unity study for COVID-19. Zenodo; 2022.

51. Ratovoson R, Razafimahatratra R, Randriamanantsoa L, et al. Household transmission of COVID-19 among the earliest cases in Antananarivo, Madagascar. *Influenza Other Respir Viruses*. Jan 2022;16(1):48-55. doi:10.1111/irv.12896

52. Reukers DFM, van Boven M, Meijer A, et al. High infection secondary attack rates of SARS-CoV-2 in Dutch households revealed by dense sampling. *Clinical infectious diseases : an official publication of the Infectious Diseases Society of America*. 2021;(a4j, 9203213)doi:<https://dx.doi.org/10.1093/cid/ciab237>

53. Ripabelli G, Sammarco ML, Cannizzaro F, Montanaro C, Ponzio GV, Tamburro M. A Coronavirus Outbreak Linked to a Funeral Among a Romani Community in Central Italy. *Frontiers in medicine*. 2021;8(101648047):617264. doi:<https://dx.doi.org/10.3389/fmed.2021.617264>

54. Salihefendic N, Zildzic M, Huseinagic H, Ahmetagic S, Salihefendic D, Masic I. Intrafamilial Spread of COVID-19 Infection Within Population in Bosnia and Herzegovina. *Materia socio-medica*. 2021;33(1):4-9. doi:<https://dx.doi.org/10.5455/msm.2021.33.4-9>

55. Semakula M, Niragire F, Umutoni A, et al. The secondary transmission pattern of COVID-19 based on contact tracing in Rwanda. *BMJ global health*. 2021;6(6)doi:<https://dx.doi.org/10.1136/bmjgh-2020-004885>

56. Shah K, Kandre Y, Mavalankar D. Secondary attack rate in household contacts of COVID-19 Paediatric index cases: a study from Western India. *Journal of public health (Oxford, England)*. 2021;43(2):243-245. doi:<https://dx.doi.org/10.1093/pubmed/fdaa269>

57. Shah K, Desai N, Saxena D, Mavalankar D, Mishra U, Patel GC. Household Secondary Attack Rate in Gandhinagar district of Gujarat state from Western India. *medRxiv*. 2020:2020.09.03.20187336. doi:10.1101/2020.09.03.20187336

58. Shambel H, Alayu M, Tayachew A, et al. Ethiopia FFX Unity study for COVID-19. Zenodo; 2022.

59. Sharma DP, Singh DMM, Rao DS, et al. India MAMC Delhi FFX Unity study. Zenodo; 2022.

60. Son H, Lee H, Lee M, et al. Epidemiological characteristics of and containment measures for COVID-19 in Busan, Korea. *Epidemiology and health*. 2020;42(101519472):e2020035. doi:<https://dx.doi.org/10.4178/epih.e2020035>

61. Soriano-Arandes A, Gatell A, Serrano P, et al. Household SARS-CoV-2 transmission and children: a network prospective study. *Clinical infectious diseases : an official publication of the Infectious Diseases Society of America*. 2021;(a4j, 9203213)doi:<https://dx.doi.org/10.1093/cid/ciab228>

62. Sreedevi A, Satheesh M, Usha A. India AIIMS Kochi FFX Unity study. Zenodo; 2022.

63. Sun WW, Ling F, Pan JR, et al. [Epidemiological characteristics of COVID-19 family clustering in Zhejiang Province]. *Zhonghua yu fang yi xue za zhi [Chinese journal of preventive medicine]*. 2020;54(6):625-629. doi:<https://dx.doi.org/10.3760/cma.j.cn112150-20200227-00199>

64. Teherani MF, Kao CM, Camacho-Gonzalez A, et al. Burden of Illness in Households With Severe Acute Respiratory Syndrome Coronavirus 2-Infected Children. *JOURNAL OF THE PEDIATRIC INFECTIOUS DISEASES SOCIETY*. 2020;9(5):613-616. doi:10.1093/jpids/piaa097

65. Telle K, Jorgensen SB, Hart R, Greve-Isdahl M, Kacelnik O. Secondary attack rates of COVID-19 in Norwegian families: a nation-wide register-based study. *European journal of epidemiology*. 2021;(ere, 8508062)doi:<https://dx.doi.org/10.1007/s10654-021-00760-6>

66. Thiel SL, Weber MC, Risch L, et al. Flattening the curve in 52 days: characterisation of the COVID-19 pandemic in the Principality of Liechtenstein - an observational study. *Swiss medical weekly*. 2020;150(d10, 100970884):w20361. doi:<https://dx.doi.org/10.4414/smw.2020.20361>

67. Tibebu S, A. Brown K, Daneman N, Paul LA, Buchan SA. Household secondary attack rate of COVID-19 by household size and index case characteristics. *medRxiv*. 2021:2021.02.23.21252287. doi:10.1101/2021.02.23.21252287

68. Tippett Barr BA, Herman-Roloff A. Kenya Table Unity Studies FFX. Zenodo; 2022.

69. Walker JG, Tskhomelidze I, Trickey A, et al. Epidemiology and transmission of COVID-19 in cases and close contacts in Georgia in the first four months of the epidemic. *medRxiv*. 2021:2021.03.22.21254082. doi:10.1101/2021.03.22.21254082

70. Wang Y, Tian HY, Zhang L, et al. Reduction of secondary transmission of SARS-CoV-2 in households by face mask use, disinfection and social distancing: a cohort study in Beijing, China. *BMJ GLOBAL HEALTH*. 2020;5(5)doi:10.1136/bmjgh-2020-002794

71. Wang Z, Ma W, Zheng X, Wu G, Zhang R. Household transmission of SARS-CoV-2. *The Journal of infection*. 2020;81(1):179-182. doi:<https://dx.doi.org/10.1016/j.jinf.2020.03.040>

72. Wei L, Lv Q, Wen Y, et al. Household transmission of COVID-19, Shenzhen, January-February 2020. *medRxiv*. 2020:2020.05.11.20092692. doi:10.1101/2020.05.11.20092692

73. Wilkinson K, Chen X, Shaw S. Secondary attack rate of COVID-19 in household contacts in the Winnipeg Health Region, Canada. *Canadian journal of public health = Revue canadienne de sante publique*. 2021;112(1):12-16. doi:<https://dx.doi.org/10.17269/s41997-020-00451-x>

74. Wu J, Huang Y, Tu C, et al. Household Transmission of SARS-CoV-2, Zhuhai, China, 2020. *Clinical infectious diseases : an official publication of the Infectious Diseases Society of America*. 2020;71(16):2099-2108. doi:<https://dx.doi.org/10.1093/cid/ciaa557>

75. Wu P, Liu F, Chang Z, et al. Assessing asymptomatic, pre-symptomatic and symptomatic transmission risk of SARS-CoV-2. *Clinical infectious diseases : an official publication of the Infectious Diseases Society of America*. 2021;(a4j, 9203213)doi:<https://dx.doi.org/10.1093/cid/ciab271>

76. Wu Y, Song S, Kao Q, Kong Q, Sun Z, Wang B. Risk of SARS-CoV-2 infection among contacts of individuals with COVID-19 in Hangzhou, China. *Public health*. 2020;185(qi7, 0376507):57-59. doi:<https://dx.doi.org/10.1016/j.puhe.2020.05.016>

77. Xie W, Chen ZH, Wang Q, et al. Infection and disease spectrum in individuals with household exposure to SARS-CoV-2: A family cluster cohort study. *JOURNAL OF MEDICAL VIROLOGY*. 2021;93(5):3033-3046. doi:10.1002/jmv.26847

78. Yadav DK, Subhashini D, Kant DS. India AIIMS Delhi FFX Unity study. Zenodo; 2022.

79. Yi B, Fen G, Cao D, et al. Epidemiological and clinical characteristics of 214 families with COVID-19 in Wuhan, China. *International journal of infectious diseases : IJID : official publication of the International Society for Infectious Diseases*. 2021;105(c3r, 9610933):113-119. doi:<https://dx.doi.org/10.1016/j.ijid.2021.02.021>

80. Yi C, Aihong W, Bo Y, et al. Epidemiological characteristics of infection in COVIU-19 close contacts in Ningbo city. *Chinese Journal of Endemiology*. 2020;41(5):667-671. doi:10.3760/cma.j.cn112338-20200304-00251

81. The World Bank. World Bank Country and Lending Groups

6th December, 2021. Accessed 6th December, 2021. <https://datahelpdesk.worldbank.org/knowledgebase/articles/906519-world-bank-country-and-lending-groups>

82. CoVariants. Overview of Variants in Countries

23rd February 2022. <https://covariants.org/per-country>

83. GISAID. <https://www.gisaid.org/>
